# Avoiding adverse events in interventional radiology – a systematic review on the instruments

**DOI:** 10.1186/s42155-023-00413-7

**Published:** 2024-01-03

**Authors:** Sophia Freya Ulrike Blum, Ralf-Thorsten Hoffmann

**Affiliations:** 1 Institute and Polyclinic for Diagnostic and Interventional Radiology, University Hospital Carl Gustav Carus, Technical University Dresden, Fetscherstraße 74, Dresden, 01307 Germany; 2 Quality and Medical Risk Management, University Hospital Carl Gustav Carus, Technical University Dresden, Fetscherstraße 74, Dresden, 01307 Germany

**Keywords:** Safety culture, Adverse events, Checklist, Simulator training, Communication, Value-based interventional radiology, IR

## Abstract

**Background:**

Avoiding AEs is a pivotal fundament for high patient safety in an efficient interventional radiology (IR) department. Although IR procedures are considered to have a lower risk than their surgical alternatives, they account for one third of all radiological adverse events (AEs) and in general, the number of AEs is increasing. Thus, measures to prevent AEs in IR are of interest.

**Methods:**

A systematic literature search was conducted via handsearch and Ovid. A structured data extraction was performed with all included studies and their quality of evidence was evaluated. Finally, data were aggregated for further statistical analysis.

**Results:**

After screening 1,899 records, 25 full-text publications were screened for eligibility. Nine studies were included in the review. Of those, four studies investigated in simulator training, one in team training, three in checklists, and one in team time-out. Eight were monocenter studies, and five were conducted in a non-clinical context. Study quality was low. Aggregation and analysis of data was only possible for the studies about checklists with an overall reduction of the median error per procedure from 0.35 to 0.06, observed in a total of 20,399 and 58,963 procedures, respectively.

**Conclusion:**

The evidence on the instruments to avoid AEs in IR is low. Further research should be conducted to elaborate the most powerful safety tools to improve patient outcomes in IR by avoiding AEs.

## Background

According to the WHO, 15% of the total hospital activities result from adverse events (AE), and 50% of all AEs are preventable [1, 2]. In hospitals, 1/10 patients are harmed by AEs; in outpatient care, even 4/10 patients experience an AE. A large recent analysis of the frequency and rate of hospital AEs showed an increase over time [2]. Avoiding AEs improves patient outcomes and enables significant savings for the health system. Thus, avoiding AEs is pivotal for high patient safety in an efficient interventional radiology (IR) department.

Overall, procedures in IR are considered to have a lower risk than surgical alternatives due to the minimally invasive approach. However, the French National Authority for Health database in radiology, AEs in IR accounted for one third of all documented AEs [3] and rapidly evolving new techniques bear a significant risk for AEs. Furthermore, 53% of all AEs were preventable [3]. For this reason, principles of avoiding AEs that successfully have been implemented in surgery were transferred to the Standards of Practice of the Society of Interventional Radiology (SIR) in 2008 [4]. Continuous professional development for all staff is vital to ensure a highly motivated and skilled workforce that provides a high-quality, safe and sustainable service. Therefore, trainee rotation through IR units and dedicated consultant time to deliver training must be part of planning [5]. Even in countries where risk management is mandatory, only a few departments have incorporated it into their routine schedules.

Consequently, chief physicians must serve as role models, actively implement safety tools in their IR departments and, most importantly, foster a positive culture of failure management [6]. Safety culture is the product of beliefs, values, competencies, and patterns of behaviour that define the organization’s overall commitment to quality and patient safety [7]. According to the literature, a strong safety culture reduces the frequency of AEs and the barrier to reporting AEs [8]. At the same time, cost savings were found after a hospital-wide patient safety strategy [9]. Most of the study results implicate an association between chief physicians’ commitment to patient safety and fewer AEs [8, 10–12].

Furthermore, chief physicians are creators of safety culture. It entails the internalization of the values and beliefs of hospital personnel. Managers strongly influence individual attitudes and behaviours toward safety, establishing an identifiable climate of work processes. Thus, safety culture should be established in daily IR practice and resident education in every IR department [13, 14].

A systematic review from 2015 summarizes the frequency of medical errors in IR [15]. According to this review, 78% of the mistakes occurred during a procedure, 12% occurred before and 10% after a procedure. Another important discovery was that 55–84% of the mistakes might be preventable in IR. These findings help to understand that safety measures are needed for every step of an interventional procedure.

Taken together, avoiding AEs follows the principles of value-based radiology [16]. Inter alia, it aims to increase patient safety which can be measured by monitoring and controlling key performance indicators representing the quality and safety of radiological services, such as rate of AEs or quality of indication. In this subspecialty of IR, the term value-based IR should be employed.

This systematic review focuses on all preventive tools to avoid typical AEs in IR. It explains risk management tools and training, as well as the quality of evidence for every tool.

## Methods

We conducted a systematic literature search via handsearch in Medline and EMBASE (via Ovid). The search strategy contained pre-defined keywords, search and MESH terms (Table 1). The Preferred Reporting Items for Systematic reviews and Meta‑Analyses (PRISMA)-Checklist was applied for reporting. After removing duplicates, all results were screened at title-/abstract and full-text level using Rayyan (https://rayyan.qcri.org/). Inclusion criteria were: full-text manuscript, focus on tools to avoid errors in IR, quantification of errors in IR and influence of tool on the error rate should be supplied in manuscript.

**Table 1:** Search strategy

Search strategy (Ovid)	
1	(team rehearsal or team time out or safety culture or checklist* or communication or simulat* or virtual*).ti,ab
2	(IR or interventional radiology or image-guided).ti,ab
3	(prevent or complication* or error or adverse event* or outcome or mistake*).ti,ab
4	1 and 2 and 3
5	Remove duplicates from 4

A spreadsheet was used to record summary data from each study: country, setting and design. All studies were aggregated according to their main study objective. To rate the quality of evidence, a structured data extraction was performed according to the Grading of Recommendations Assessment, Development, and Evaluation (GRADE) system [17, 18]. Data recorded for each study included the number of interventional procedures and total errors before and after implementing security measure. From these aggregated data, mean, range, median and interquartile range (IQR) were calculated across all studies for the total error rate per procedure (total errors divided by number of procedures).

## Results

**Fig. 1:**
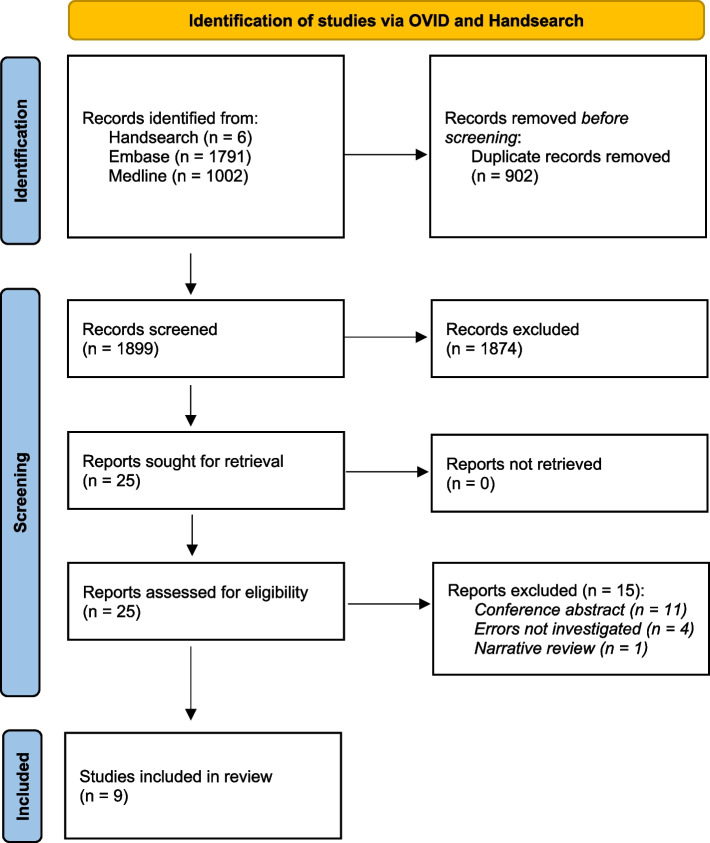
PRISMA 2020 flow diagram for new systematic reviews which included searches of databases

After screening 1,899 records, 25 full-text publications were screened for eligibility (Fig. 1). The most exclusions were conference abstracts (*n* = 11). Nine studies met the inclusion criteria and were included in the review [19–27]. Study characteristics are given in Table 2, with 8/9 being monocenter studies and only four studies in a clinical setting. Four studies investigated in simulator training, one in team training, three in checklists, and one in team time-out.

**Table 2:** Study characteristics in chronological order

Study	Country	Setting	Study type
Morbi et al. (2012) [24]	United Kingdom	Clinical, vascular interventional radiology	Interventional study design, monocenter
Fargen et al. (2013) [25]	USA	Clinical, neurointerventional procedures	Interventional study design, monocenter
Lutjeboer et al. (2015) [26]	Netherlands	Clinical, elective IR procedures	Prospective, monocenter
Cates et al. (2016) [19]	USA	Simulated angiography suite	Prospective, monocenter, blind, randomized
Nawka et al. (2020) [20]	Germany	Simulated angiography suite	Prospective, monocenter
Zaika et al. (2020) [21]	Canada	Workstation with haptic feedback simulator	Prospective, monocenter
Ramjeeawon et al. (2020) [23]	United Kingdom	Simulated angiography suite	Prospective, monocenter
Siewert et al. (2022) [27]	USA	Clinical	Retrospective, multicenter
Zaika et al. (2023) [22]	Canada	Workstation with haptic feedback simulator	Prospective, monocenter

### Simulator training

Four studies investigated the effect of training on the occurrence of AEs or indirect measures for AEs [19–22]. They all had a prospective study design, focussed on neurointerventional procedures and were limited due to small sample sizes. Only one study had a randomized and blinded approach and was conducted in a clinical setting [19]. The other three studies were conducted in an in vitro setting. Although they had a small sample size, a significant effect was found. Two of the three in vitro studies included medical students as study subjects [21, 22], whereas the other two studies compared interventional radiologists [19, 20].

### Team training

One study investigated team training in IR with a prospective, in vitro approach [23]. In the comparison of team training during two simulations of emergency thoracic endovascular aortic repair, the authors could not confirm a reduction in technical errors after the provision of team training [23].

### Checklists

For IR, there is only a small body of evidence that checklists reduce AEs [25–27]. One study used an interventional study design [25], one was prospective [26], and another one was retrospective [27]. Two studies investigated the effect of pre-procedural checklists [25, 26], and one study focused on a post-procedural checklist [27].

As part of an institutional quality improvement project, Fargen et al. showed an overall reduction in AE rates, which had been low already, and an improvement in communication in interventional neuroradiology with the help of a dedicated checklist [25].

In one retrospective study about the effect of a post-procedural checklist, the authors showed a significant reduction of AEs and found a decrease in repeat procedures after implementing the post-procedural checklist [27]. This recent study, comprising a large number of procedures, was based on a self-reporting system for AEs [27].

### Team time-out

To date, there is one investigation on the effectiveness of a team time-out in IR. In a single-center study in vascular IR, preventable failures and failure rates per hour decreased significantly with the implementation of a preprocedural team time-out. The authors showed that 11% of the AEs were due to communication errors [24].

### Quality of the studies

All studies and their quality according to the GRADE system are given in Table 3. The quality of the studies was limited due to the low number of procedures, inaccurate reporting of results and the monocenter approach.

**Table 3:** Summary of findings table and GRADE evidence profile for safety tools in IR. The study design is already given in Table 2 and was included in the assessment of the quality

Study	Study subject	N	Effect	Outcome measure	Limitation	Quality according to GRADE
**Simulator training**						
Cates et al. (2016) [19]	Intraoperative errors for carotid artery angiography	6 study subjects (simulator trained operators) vs. 6 controls (traditional training)	49% less intraoperative errors (*p* < 0.001)	Objectively classified intraoperative error	Small sample size	High
Nawka et al. (2020) [20]	Dangerous maneuvers in 3 different aneurysm models	3 experienced vs. 3 inexperienced operators	Less dangerous maneuvers in experienced group (median 0.0; 0.0–1.0 IQR) vs. inexperienced group (1.0; 0.0–1.5) (*p* = 0.014)	Dangerous maneuvers^a^	In vitro study, small sample size	Very low
Zaika et al. (2020) [21]	Time spent in incorrect vessel in simulation of R-MCA aneurysm	8 clinical anatomy graduate students and 6 residents in neurosurgery and radiology specialties	Significant drop of time spent in incorrect vessel over 8 sessions (*p* < 0.05)	Pre-defined errors (any deviation from correct pathway)^a^	In vitro study, small sample size	Very low
Zaika et al. (2023) [22]	Coiling mistakes in simulation of R-MCA aneurysm	12 participants with minimal or no knowledge of endovascular skills and basic vascular background	Improvement after 6 sessions, but not statistically significant	Coiling errors (protrusion, perforation)^a^	In vitro study, small sample size	Low
**Team training**						
Ramjeeawon et al. (2020) [23]	Errors during simulation of TEVAR before and after team training	One team simulation before and after training	No decrease of errors (*p* = 0.109)	Pre-defined errors	In vitro, small study sample	Low
**Checklist**						
Fargen et al. (2013) [25]	Number of adverse events or near-misses before/ after implementation of a checklist in a neurointerventional department	71 procedures before vs. 60 after implementation of checklist	Significant reduction of total number of adverse events or near-misses (*p* = 0.001)	Adverse events or near-misses		Low
Lutjeboer et al. (2015) [26]	Number of process deviations in pre-procedural planning and sign in for IR procedures when performing an appointment prior to procedure	110 controls vs. experimental group	Reduction of mean number of process deviations from 0.39 to 0.06 (*p* < 0.001)	None	EVAR and neuro-interventions were excluded	Very low
Siewert et al. (2022) [27]	Evaluation of effects after implementation of a postprocedural checkout list	34 safety reports	Reduction of AEs (0.069% to 0.034%; 43% decrease, *p* = .05) Reduction of repeat procedures (0.040% to 0.007%; 80% decrease, *p* = 0.003)	Rate of AEs and repeat procedures	Very short pre-implementation period	Low
**Team Time Out**						
Morbi et al. (2012) [24]	Number of failures before/ after implementation of a preprocedural team rehearsal for vascular interventional procedures	55 procedures before and 33 after implementation of preprocedural team rehearsal	Decrease of preventable failures (54.6% vs. 27.3%) and failures per hour (18.8 vs. 9.2) (*p* < 0.001 for both)	Pre-defined failures	Assessed by a medical student with no prior technical knowledge	Low

*Abbreviations*: *IQR* interquartile range, *(T)EVAR* (thoracic) endovascular aortic repair, *R-MCA* right middle cerebral artery

^a^Indirect measure for patient outcome

### Aggregated total error reduction

Table 4 shows the total error reduction per procedure over all studies. Only the publications about checklists could be aggregated for further analysis. An aggregation of the four studies about simulator training was not possible due to unprecise reporting of the data with missing total numbers of errors and procedures.

**Table 4:** Total error rates before and after implementation of checklists. IQR: interquartile range

Before checklist implementation				After checklist implementation			
Total number of errors	Total number of procedures	Total error rate per procedure		Total number of errors	Total number of procedures	Total error rate per procedure	
		Mean (range)	Median (IQR)			Mean (range)	Median (IQR)
82	20,399	0.004 (0.001–0.35)	0.35 (0.18–0.37)	33	58,963	0.001 (0.0003–0.1)	0.06 (0.03–0.08)

## Discussion

This systematic review found only nine studies on safety measures in IR. So far, our toolkit to avoid AEs consists of the periprocedural checklist, simulator training, team time-out, and team training. As the quality of the studies could be better, we observed a need for more evidence for the efficacy of all these measures. Altogether, there is a need and considerable potential for further research on safety measures in IR.

The Quality in Australian Health Care Study reported that one third of all AEs were a failure in the technical performance of an indicated procedure or operation [28]. Data about IR does not exist. There is a learning curve to every procedure, as shown in adrenal venous sampling by Jakobsson et al., where the technical success rate rises from 65% in the first year to a stable success rate above 90% after seven more years [29].

Four studies covering the effect of simulator training on AEs in IR were identified in this review. A small analysis of neurointerventional skills in simulator training reported significantly more dangerous manoeuvres by inexperienced operators than experienced ones [20]. Simulators give the chance to analyse and specifically reduce such dangerous procedures before performing them on patients. Accordingly, another small series of simulation training for cerebral angiography showed a significant reduction in navigational errors after eight sessions [21]. The same research group recently reported fewer perforations and coil misplacements in a small series of simulator training by novice medical students [22]. The effect of virtual training was shown for carotid angiographies in a small prospective clinical trial by Cates and colleagues. They found significantly lower intra-operative errors when comparing standard-trained and virtual reality-trained operators [19].

Although all mentioned studies showed improvements in the number of errors in a real or in vitro environment, there is no investigation measuring the direct influence of simulator training on patient outcomes. Moreover, there is a focal point on neurointerventional IR.

In the light of optimal patient care, it seems reasonable to train interventions with a simulator with the possibility to achieve a high success rate and a very low rate of complications at the same time. Therefore, fundamental and high-risk interventions and infrequent AEs should be part of the training [30]. These training sessions should be analysed and reflected. A critical component of those training sessions is the environment where the intervention is usually performed, including the procedural team. Also, experts can train new or complex interventions before performing them in real, maybe even experiencing or simulating mistakes or AEs in vitro [30]. Eventually, simulator trainings bear the potential to increase learning curves also for experienced operators in very complex and infrequent interventions. No clinical study has investigated the potential to increase patient outcomes through simulation or virtual reality training.

This review identified one in vitro study about the effect of team training on reducing AEs in IR, which did not suggest a substantial reduction of AEs. Team training is established across a broad spectrum of medical disciplines, usually taking place on-site at the workplace and requiring 4–6 h in most cases [31]. They primarily target situational awareness, communication, leadership, and role clarity in crisis resource management. Every participant in this training has the opportunity to update their skills in a safe multidisciplinary setting with a team of 5–6 trainees [31]. A decisive advantage of team training is the possibility of identifying potential errors and correcting them before they happen. Despite the low evidence in IR, efforts should be undertaken to plan team training as the positive effect of team training on patient outcomes is known from a various medical fields such as surgery, obstetrics, operating room, paediatrics, and pediatric intensive care unit [32]. Especially time-critical emergency IR procedures such as resuscitative endovascular balloon occlusion of the aorta or emergency percutaneous endovascular aortic repair might profit from team training. Notably, the sustainability of team training is not clear until now. Some authors reported sustained improvements even 12 months after training. Others observed only short-term improvements, implying to undergo team training regularly [32]. Altogether, a promising approach would be the evaluation of AEs in a real clinical setting for IR teams before and after carrying out training together.

Checklists are an inevitable instrument to increase patient safety during surgical procedures and to save time. Three studies about the effects of checklists on AEs in IR were found in this review. The aim of checklists is a structured and complete patient preparation and planning on the day before intervention. Furthermore, there is proof that checklists can decrease the number of postponed interventions and significantly reduce non-conformance within the procedures [33]. While surgical disciplines use a checklist in 90% according to a representative survey [34], only 48% of interventionalists use a checklist for all interventional procedures (computed tomography, ultrasound, fluoroscopy, stereotactic biopsy, angiography), with a focus on angiographic interventions according to the publication [35].

In contrast to this data, physicians prefer to work with checklists when asked about their relevance. Moreover, they expect a better awareness of patient safety and a higher efficacy [33]. None of the studies published the checklist compliance bearing an unfavourable bias for checklists. One study was based on a self-reporting system for AEs, which might lead to an underestimation of errors [27].

Corso et al. found adherence to checklists in 64.5% before starting a safety and quality program in an interventional department, rising to 84.4% after the program [36]. These data are equally found for surgical checklists with a 90% compliance rate and 61% completion rate [34]. Typical barriers to the completion of checklists were duplication of items within existing checklists, poor communication between surgeon and anaesthetist, time spent completing the checklist for no perceived benefit, and lack of understanding and timing of item checks, ambiguity, unaccounted risks and a time-honoured hierarchy. For this reason, the authors propose the adoption of surgical checklists [34]. Accordingly, it is highly recommended to adjust the CIRSE checklist [37] to the individual situation of every department, to audit the compliance and completion rates and to re-evaluate the contents continuously. Importantly, periprocedural checklists do not necessarily aid teamwork and communication.

An essential finding of this review is the total error reduction per IR procedure over all studies investigating the influence of checklists on AEs. Specifically, his review found a 4-fold mean decrease and a 6-fold median decrease of errors when checklists are used in IR. According to a review about checklists in surgery by Treadwell et al., 30-day-mortality was 15% less likely, a surgical site complication was 70% less likely and surgical complications were 55% less likely when checklists were used [38]. Altogether, this considerable impact on patient safety leads to a strong recommendation to use periprocedural checklists in IR.

One study was identified in this review, showing a significant reduction of AEs by implementing team time-out. As part of the checklist, the team time-out is the last verbal synchronization directly before starting the intervention. It underlies the doublecheck principle and is a tool to avoid wrong site or wrong patient intervention and exposure to known allergens, common avoidable errors in IR [39]. Data only focusing on the team time-out process in other medical disciplines is rare. A neurosurgical study reported similar effects of an extension of the surgical checklist by a team time-out, significantly reducing errors [40].

According to The Joint Commission, team time-out is an effective tool to avoid serious reportable events, also known as never events. They are defined as serious and harmful, largely preventable clinical events [41]. Important examples are interventions on the wrong site, wrong patient, or wrong procedure. The Universal Protocol for Preventing Wrong Site, Wrong Procedure, Wrong Person Surgery from The Joint Commission was applied to the Quality Improvement Guidelines for Preventing Wrong Site, Wrong Procedure, and Wrong Person Errors by the SIR. For the practical implementation of team time-out, they state that it must be done immediately before the invasive procedure and in the location where the procedure takes place. Aside from that, the entire team must be involved in the process [4].

What has yet to be addressed in studies so far? Communication standards and clinical case discussion were not evaluated in the studies. Aviation has a long and successful history of this facet of risk management and quality improvement. Aviation and medicine involve people working in highly complex systems so that this knowledge can be transferred to medicine. Pilots are open and committed to discussing any event to improve the system's safety. Their superiors support them without fear of punishment or retribution. Although senior operators are the decision-makers, they must encourage open communication. In aviation, it is common practice to focus all communication during critical portions of a procedure.

Similarly, there should be no non-essential communication during an intervention or other disrupting background noise, and feedback on errors must be possible [42]. Communication errors can occur at any level of patient care [39]. There is a high need for communication training for staff in the IR suite.

There is currently no study on the reduction of errors or even improvement of patient outcomes by the adoption of communication standards other than team time-outs and clinical case discussions.

Device misuse or malfunction is a preventable AE. No studies observing the effect of stringent device instruction on the rate of AEs and patient outcomes are currently available. In a large retrospective study, Dagli and colleagues found that device misuse or malfunction accounted for 15% of all preventable AEs identified [43]. Data on device-related AEs are sparse. A review of the Manufacturer and User Facility Device Experience (MAUDE) database during percutaneous nephrolithotomy found that device malfunction was caused by misuse by the operator in more than half of the cases [44]. Device-related causes for serious AE were also registered in the French National Authority for Health database [3]. Adequate device instruction, therefore, might have great potential for significant improvement in patient safety. Additionally, the beforementioned list of serious reportable events also contains AEs potentially occurring in IR, such as unintended retention of a foreign object in a patient, patient death or serious injury associated with the misuse or malfunction of a device and intravascular air embolism [41].

Overall, studies about avoiding AEs in IR are sparse, although accreditation requirements contain the usage of specific instruments to pertain to patient safety [45]. IR can benefit from the longstanding experience of other medical disciplines and incorporate established tools in their routine process. Nonetheless, more scholarly reappraisal is needed to identify the most effective tools to avoid AEs in IR, characterized by its less invasive nature, a large variety of procedures, and high throughput. Specific study designs are needed to assess the effects of tools to prevent AEs, as they are relatively rare [46]. Suitable for this case is a before and after design in a clinical setting, ideally on a national scale, e.g., via interventional data registers accompanied by accreditation audits.

## Conclusion

In conclusion, the evidence on the instruments used to avoid AEs in IR is low. Communication skills have yet to be in the scope of studies. However, the first results are promising and similar to surgical disciplines, where most measures are firmly established. Further research should be conducted to elaborate on the most powerful safety tools to improve patient outcomes in IR by avoiding AEs.

## Data Availability

The datasets used and/or analysed during the current study are available from the corresponding author on reasonable request.

## Abbreviations

AE: Adverse Event
GRADE: Grading of Recommendations Assessment, Development, and Evaluation
IQR: Interquartile range
IR: Interventional Radiology
PRISMA: Preferred Reporting Items for Systematic reviews and Meta‑Analyses
